# Rat bite fever with osteomyelitis and discitis: case report and literature review

**DOI:** 10.1186/s12879-021-06172-x

**Published:** 2021-05-26

**Authors:** Steven H. Adams, Rahul Mahapatra

**Affiliations:** 1 Upstate Medical University, 750 E Adams St, Syracuse, NY 13210 USA; 2 Department of Medicine, Division of Infectious Diseases, Upstate Medical University, Syracuse, NY USA; 3 Upstate Infectious Diseases Associates, 725 Irving Ave, Suite 311, Syracuse, NY 13210 USA

**Keywords:** Rat bite fever, Vertebral osteomyelitis, Discitis, Case report

## Abstract

**Background:**

Rat bite fever (RBF) is a rare systemic febrile illness transmitted by rats. *Streptobacillus moniliformis* is a pleomorphic Gram-negative bacillus which is the usual etiologic organism for rat bite fever in the United States.

**Case presentation:**

Here we present a case of rat bite fever complicated by vertebral osteomyelitis and discitis. The patient revealed an exposure history of being bitten by pet rats. The patient’s symptoms dramatically improved with a six-week course of cephalexin therapy.

**Conclusions:**

It is important to obtain a thorough zoonotic exposure history and maintain rat bite fever in the differential when considering potential causes of discitis and osteomyelitis.

## Background

Rat bite fever (RBF) is an uncommon systemic febrile illness transmitted by rats. *Streptobacillus moniliformis* is a pleomorphic Gram-negative bacillus which is the usual etiologic organism for RBF in the United States. Osteomyelitis and discitis have very rarely been reported in association with RBF.

## Case presentation

A 55-year old male presented to an academic medical center in February 2020 with a six-week history of increasing midline back pain. He had a history of chronic mid- and lower back pain as the result of degenerative disc disease, however his pain had abruptly worsened in the 6 weeks prior to presentation. Pain was worsened with trying to sit up straight or walk. Pain was partially relieved with acetaminophen, ibuprofen, and oxycodone 5 mg tablets taken as needed. He reported no associated fevers, chills, or night sweats. He did not recall a febrile illness prior to the onset of symptoms. He did report anorexia and 15-pound weight loss in the last 6 weeks. His medical history included chronic obstructive pulmonary disease, chronic hepatitis C infection, hyperlipidemia, generalized anxiety disorder, and lumbar degenerative disc disease. His surgical history was notable for cervical spine laminectomy in the remote past. His social history was notable for heavy and ongoing tobacco use with a 60 pack-year smoking history. He denied alcohol or illicit drug use including intravenous drug use.

He was afebrile and hemodynamically stable on presentation. Physical exam revealed a gaunt Caucasian male in moderate distress due to pain. Poor dentition was noted. His cardiopulmonary examination was unremarkable. On spinal examination, no bony tenderness was elicited upon palpation of the thoracic and lumbar spine, however paraspinal tenderness was noted in the lumbar spine. Neurologic examination including strength and sensation of the lower extremities was intact. Babinski reflex was downward bilaterally.

**Fig. 1:**
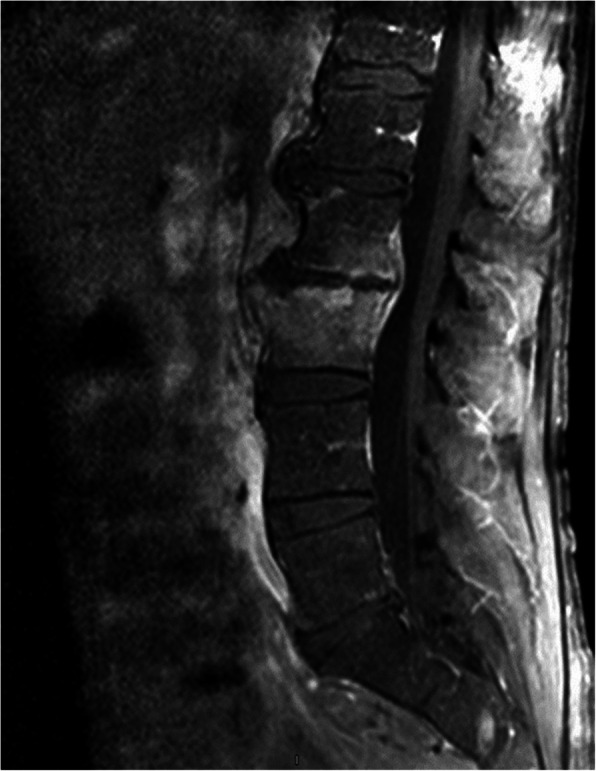
MR lumbar spine with contrast, T1WFS: There is abnormal enhancement of the lower endplate of L2 vertebral body and there is diffuse enhancement of most of L3 vertebral body with irregularity of the upper endplate. The abnormal enhancement slightly extends into the intervertebral disc. There is a thickening in the anterior epidural space and the central and right paracentral region at L2–3 level with a possibility of a tiny pocket of nonenhancement within the enhancing anterior epidural space measures about 2 × 4 mm which could represent a small epidural abscess or hematoma from the recent trauma

MRI with and without gadolinium contrast revealed abnormal enhancement of the lower endplate of the L2 vertebral body as well as diffuse enhancement of the L3 vertebral body with irregularity of the upper endplate. This abnormal enhancement extended to the intervertebral disc. Findings were suggestive of discitis with osteomyelitis not excluded. Additionally, there was enhancement and thickening in the anterior epidural space measuring 2 mm × 4 mm which may represent epidural abscess or hematoma (Fig. 1). Laboratory evaluation including CBC with differential and comprehensive metabolic panel was unremarkable. Sedimentation rate was 36 mm/hr. and C-reactive protein was 30.1 mg/L. Blood cultures were sterile.

**Fig. 2:**
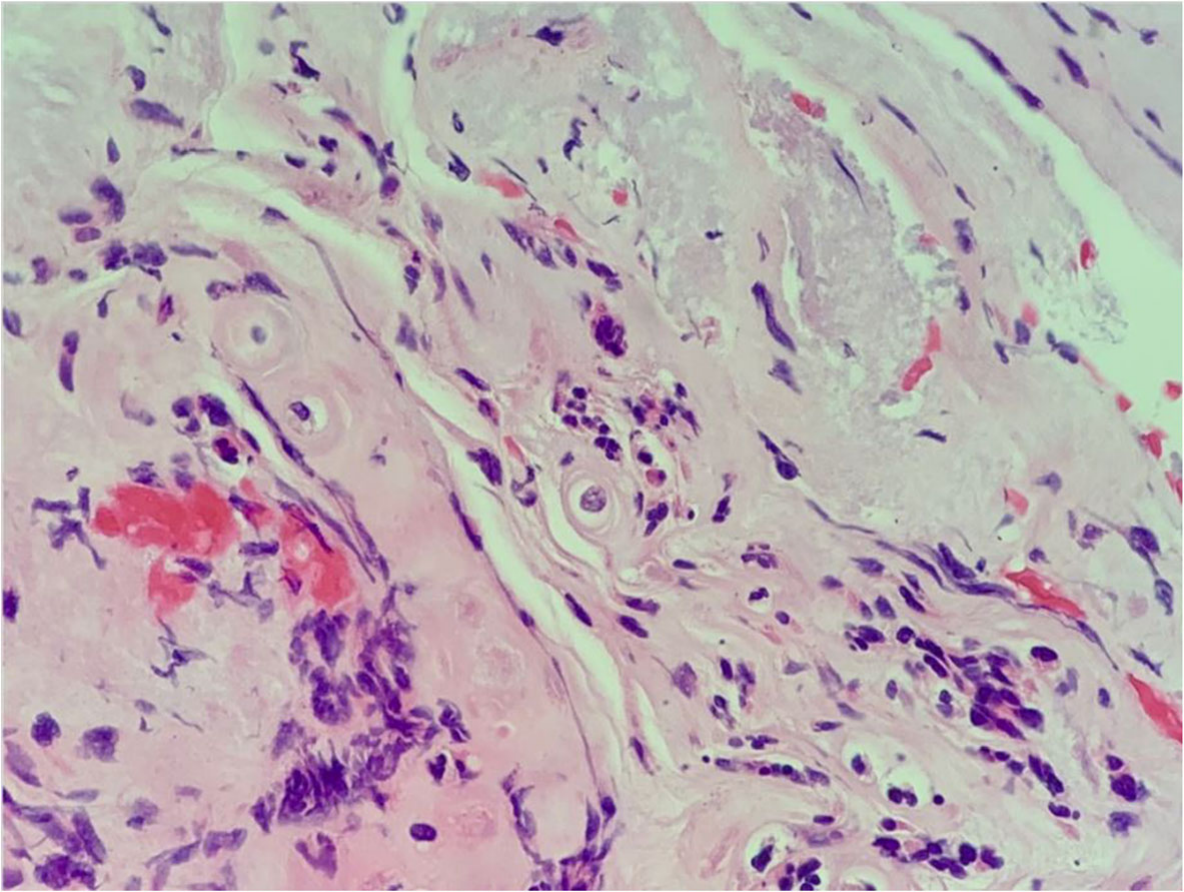
H&E stain, vertebral disc fibroelastic cartilage infiltrated by acute inflammation, consistent with discitis

Based on initial results of clinical, laboratory, and radiographic evaluation, antibiotic therapy was withheld. CT-guided aspiration of the L2-L3 disc was performed for culture and histopathology. Gram stain revealed Gram variable rods. Histopathology revealed fibrocartilage with degenerative changes and acute inflammation suggestive of discitis (Fig. 2). He was seen in Infectious Diseases outpatient consultation, initially offered intravenous antibiotic therapy however patient requested oral antibiotic therapy for empiric treatment of discitis. He was started on treatment with cephalexin 1 g by mouth three times a day as well as linezolid 600 mg by mouth twice a day. (Initially, prior to the revelation of RBF infection, linezolid was chosen (in addition to cephalexin) because Gram stain revealed Gram-positive rods. The intention was to cover Corynebacterium spp. and coagulase-negative staphylococci, skin flora which tend to have resistance to beta-lactams but susceptibility to vancomycin or linezolid.) Cultures were sent to a reference laboratory, with growth noted on Mueller-Hinton media with 5% sheep blood. The organism was identified as *Streptobacillus moniliformis* by matrix-assisted laser desorption ionization time-of-flight mass spectroscopy (MALDI-TOF). On further history, it was revealed that patient had two pet rats and had sustained numerous bites in the last 1 year prior to symptom onset. In vitro susceptibility testing using broth microdilution revealed low MIC for penicillin (< 0.06 μg/ml), ampicillin (< 0.12 μg/ml), and ceftriaxone (< 0.06 μg/ml), with elevated MIC > 4 μg/mL for gentamicin. The patient’s symptoms dramatically improved with cephalexin so a decision was made not to switch to oral or IV penicillin. Linezolid was discontinued after 2 weeks once culture results were available. He completed 6 weeks of oral cephalexin therapy with dramatic improvement of back pain. The patient was offered TEE to evaluate for endocarditis. However, he declined to have the test done as this coincided with the onset of CoVID-19 pandemic.

## Discussion

Rat bite fever (RBF) caused by *Streptobacillus moniliformis* is transmitted to humans predominantly through rodent bites and scratches. *S. moniliformis* has been commonly detected in wild and pet rats [1]. A diverse array of animals has been implicated to harbor, or suffer from, *S. moniliformis* infections, such as gerbils, squirrels, turkeys, koalas, macaques, dogs, cats, weasels, and ferrets. However, more recent metagenomic data have suggested that streptobacilli in these other rodent species may in fact be separate species altogether [1]. Moreover, novel *Streptobacillus* species (*S. hongkongensis, S. felis*) have been recently described in clinical human and veterinary infections [2].

RBF has historically been a disease of poverty. While prior to 2007 only a small minority (5%) of RBF cases had known pet rat exposure, since 2010 most cases of RBF have been linked to pet rats [3, 4]. This trend is likely attributable to the increasingly popular practice in the United States of keeping rats as house pets [5]. The actual incidence of rat bite fever is unknown as it is not a mandatory reportable disease [4].

*S. moniliformis* is a pleomorphic, non-acid-fast, Gram-negative, facultative anaerobe bacillus which tends to grow in long intermittently-beaded filaments. Difficult to grow in culture, *S. moniliformis* requires specific growth and incubation conditions [3]. Definitive identification is made by MALDI-TOF [1]. In our case, the isolate was sent to ARUP reference laboratory (Salt Lake City, UT), and species identification was made using MALDI-TOF. It must be mentioned that commercial MALDI-TOF databases do not contain spectra of all *Streptobacillus* species and that it is plausible that MALDI-TOF may not be able to differentiate between *S. moniliformis* and other *Streptobacillus* species [1]. Many hospital laboratories do not have the capabilities for accurate detection of the organism, and therefore diagnosis may be delayed [4]. Exposure to rats may often not be clarified from patient history until after *S. moniliformis* is identified by laboratory testing.

A 2007 review of 65 RBF case reports showed that clinical symptoms are fever (92%), arthralgias (66%), and rash (61%). Nausea and vomiting (40%), headache (34%), and sore throat (17%) were also common. White blood cell count was on average 12.2 10^3^/μL; only 5 patients demonstrated leukocytosis higher than 15 10^3^/μL [3]. Interestingly, these common clinical manifestations were all absent in our patient. (WBC remained under 8 10^3^/μL throughout the patient’s clinical course). It is possible that patient’s history of chronic hepatitis C infection may have blunted the patient’s adaptive and innate immune response to *S. moniliformis* infection and thereby led to the atypical clinical presentation.

Reported complications associated with RBF are extensive and include meningitis, mastoiditis, interstitial pneumonia, periarteritis nodosa, pancreatitis, pericarditis, myocarditis, hepatitis, prostatitis, septic arthritis, and abscess formation in various organs [4, 6]. Infective endocarditis due to RBF has a particularly poor outcome with mortality rates of 50% [4, 7].

The standard for treatment for *S. monoliformis* is penicillin G [4]. However, our patient was not switched to penicillin once RBF infection was revealed given his significant clinical improvement with cephalexin.

We performed a literature review for RBF cases with osteomyelitis or discitis. PubMed was searched for the following terms: *Spirillum minus*, *Streptobacillus moniliformis*, *Haverhillia multiformis*, rat bite fever, AND osteomyelitis, discitis. Our literature review uncovered associated with *S. moniliformis*, only 4 cases of osteomyelitis, 1 of possible osteomyelitis, and 4 of discitis, reported globally [6–14]. It is difficult to judge in our case whether the findings on MR imaging represent discitis or rather extension of infection from the L2-L3 endplate. Nonetheless, this is an unusual and rarely reported complication of RBF [13].

Table 1 summarizes nine cases of RBF associated with osteomyelitis or discitis, all reported from developed nations from 2008 through 2019. Ages ranged from 22-months to 80-years, with 4 females and 5 males. In only seven of the nine cases did the patient acknowledge direct exposure to rats. Four kept rats in their home for reasons including having house pets and a reptile food source. Affected joints included the cervical, thoracic, lumbar, sacral vertebrae and intervertebral discs, as well as hip, ankle, and sternoclavicular joints. Two cases required surgical debridement, while six resolved with antibiotic therapy alone (Table 1). A recent analysis of rat bite fever diagnosis in the United States reveals that the majority of encounters occur in persons aged 0–19 years [15]. Our case highlights the need to consider the diagnosis in older adults as well.

**Table 1:** Literature review summary of nine RBF cases associated with osteomyelitis or discitis, 2008 through 2019

Year	Country	Study	Age/sex	Exposure	Clinical history and findings	Significant biochemical findings	Site of osteomyelitis/discitis	Identification method of ***Streptobacillus moniliformis***	Cultures	Imaging findings	Surgical treatement	Histological findings	Diagnosis	Antibiotic treatment	Outcome
##	France	Dubois et al.	80-year/male	Rooster scratch	History: One week of shaking chills and back pain radiating to both legs on awakening. The pain subsided by time of presentation. On exam: Afebrile. Physical examination was without specific signs. Over following days developed disorders of consciousness and fever (Tmax 39 °C).	WBC 19, neutrophil count 18, CRP 488 mg/liter, fibrinogen 8.9 g/liter, procalcitonin 13 ng/ml. Later, CRP level lowered to 240 mg/liter, then to 163 and 115 mg/liter.	T5-T6 and L2-L3	16S rRNA PCR assay from psoas abscess fluid	Blood specimens inoculated into paired aerobic and anaerobic bottles gave positive results after 1–5 days. Gram-staining of psoas abscess and blood smears showed pleomorphic fusiform gram-negative rods.	CT thorax revealed pericardial and pleural effusions. CT abdomen showed right iliac psoas abscess communicating with a prosthesis screw. Bone scan showed increased signal at L3. MRI lumbar spine revealed psoas abscess and of spondylodiscitis at T5 and T6, and at L2 and L3. (Imaging details not provided).	none	n/a	Spondylodiscitis and psoas abscess	Began with empirical antibiotic therapy parenteral amoxicillin-clavulanic acid (1 g, Q8H) and ofloxacin (200 mg, Q12H). Switched to imipenem-cilastatin (1 g, Q12H), ciprofloxacin (400 mg, Q12H), and teicoplanin (600 mg, QD). Then an additional 9-week treatment with i.v. ofloxacin (200 mg, Q12H), i.v. clindamycin (600 mg, Q8H), and metronidazole (500 mg, Q8H).	In good health at 8 month follow-up.
##	United States	Flannery et al.	22-month/male	Two pet rats	History: Two days of URI symptoms, then 5 days of fever, malaise, with a worsening and blistering rash on all extremities, including palms and soles. Irritability. On exam: Mild hypertension, tachycardia, T 38.0 °C. Scattered tender, erythematous, pustular rash on hands, feet, ankles without joint swelling or tenderness on initial exam. Fevers persisted for several days with worsening rash and pain. On day 5, refused to bear weight on feet; pain with right-hip range of motion exam.	Day 1: WBC 10,200/μl, Hb 10.9 g/dl, plt 217,000/μl Day 5: WBC 18,100/μl (neutrophilic predominance), Hb 10.0 g/dl, plt 523,000/μl, CRP 5.4 mg/dl, ESR 94 mm/h.	Right-hip joint	16S RNA sequencing and DNA mapping	Blood, left-foot pustule fluid, synovial fluid, and femoral bone cultures grew Gram-negative rods (though bone culture may have been contaminated).	Ultrasound of right hip revealed joint effusion. MRI: bone marrow edema in the right proximal femoral epiphysis with edema in the surrounding muscles and fascial planes (possibly post-op changes)	Open irrigation and debridement of hip joint	n/a	Septic arthritis and possible osteomyelitis	Started on vancomycin and ceftriaxone for empirical bacterial coverage. Switched to i.v. penicillin (250,000 units/kg of body weight/day divided every 4 h). Total of 8 weeks of antibiotics, with 4 weeks of intravenous penicillin and 4 weeks of oral amoxicillin.	At one-month follow-up patient remained afebrile with normal inflammatory markers. X-rays of hips and pelvis were normal. Improved weight bearing with physical therapy. Thereafter lost to follow-up.
##	United Kingdom	Adizie et al.	29-year/male	Owner of three rats	History: Five days of malaise, feverishness, headache, sore throat, joint swellings with rash. On exam: Pustular, maculopapular and petechial rash of the extremities including palms and soles. Right knee and left ankle effusions, right second MCPJ swelling.	CRP 211, ESR 36, ferritin 417, neutrophils 7.89	Left ankle	16S rRNA PCR molecular sequencing	Blood cultures and joint aspirate were initially negative. Repeat joint aspirate showed *S. moniliformis* on 16S rRNA molecular testing.	Left ankle MRI: considerable marrow edema, moderate thick walled effusion consistent with septic arthritis and associated osteomyelitis.	none	Rash skin biopsy: mild non-specific perivascular inflammation within the subcutis	Septic arthritis and associated osteomyelitis	Initially treated with a broad spectrum antibiotic; changed to i.v. benzylpenicillin; then switched to oral penicillin after 2 weeks.	Good recovery
##	Japan	Nei et al	72-year/female	Denied any direct contact with rodents. Possibility of contact with contaminated water and/or food.	History: 8 days of fever and chills. On exam: T 38 C. Subsequent worsening severe lower back pain which limited ability to ambulate.	WBC 13.3 (95% neutrophils), alkaline phosphatase 1035 IU/L, γ-glutamyl transferase 239 IU/L, CRP 26.92 mg/dL	L3-L4 vertebrae and intervertebral disc	16S rRNA genotyping	At 2 days of incubation in aerobic culture with 5% CO2 on 5% sheep blood agar, highly pleomorphic, filamentous gram-negative bacilli are visualized. Colonies described as very tiny, transparent and slightly white.	MRI: Vertebral bodies L3 and L4 with low signal on T1WI, high signal on STIR. Low intervertebral disk with linear T2 high signal. Vertebral endplates at L3, L4 were destroyed with visible high-signal-intensity bone marrow edema.	none	n/a	Vertebral spondylodiscitis	Initially treated with cefazolin (2.0 g, Q8H) and NSAIDs. Switched to ampicillin (2.0 g/every 6 h). Switched again to sulbactum/ampicillin (3.0 g/Q6H) due to failed antimicrobial susceptibility tests.	Gradual improvement of lower back pain; gradual recovery of exercise and walking capacity. Discharged on 71st day of hospital stay.
##	Japan	Sato et al.	52-year/male	Rats infestation in his home; suspicion of bite during sleep.	History: Four days of diffuse arthralgias beginning in knees and back. Found immobile due to severe arthralgia and was taken to hospital. On exam: Afebrile, HR 110, RR 24. Scars on his fingers and feet. Warm, swollen, and tender joints with pain with passive motion. Tenderness at L5/S1 vertebrae. Day 4 of admission: systolic fell to 70 mmHg, septic shock.	WBC 10,300/mL (88% neutrophils). Creatinine kinase 789 U/L. CRP 34.6 mg/dL.	L5, S1 vertebrae; L5-S1 disc	MALDI-TOF MS suggested *S. moniliformis* DSM 12112 T (score value was 1.588 – unreliable). 16S rRNA molecular sequencing.	Blood cultures positive for gram-negative bacilli at 25 h/ 35 C/ 5% CO2.	T2-weighted MRI: high signal intensity in L5 and S1, destruction of L5-S1 disc space supporting diagnosis of diagnosis of vertebral osteomyelitis	Surgical debridement. Cultures from site were negative.	n/a	Vertebral osteomyelitis	Ceftriaxone, 1 g per 24 h, 6 weeks	Complete resolution of arthralgia and back pain; no long-term sequelae.
##	Canada	Akter et al.	46-year/female	Pet rat scratch	History: One-week of fever and symmetric polyarthritis of the distal extremities with morning stiffness. One day nausea, vomiting, and diarrhea. On exam: Day 1 of presentation: T 38 °C, HR 130 beats/min, BP 96/64 mmHg. Effusions in wrists, ankles, and MTPJ. Day 2 of presentation: T 39 °C. Worsening synovitis, new onset lumbar spinal pain.	Day 1: WBC 11.1, ESR 76 mm/hr., CRP 149 mg/L. Day 2: AST 105, ALT 114, ESR 124, CRP 170.	L5-S1 intervertebral disc	Cultures. (Further info not reported).	Initial blood culture was negative. Repeat cultures grew *S. moniliformis.* Right ankle synovial fluid sample culture negative.	MRI lumbosacral spine: enhancement of the vertebral end plates; T1WI showed markedly reduced signal at the L5-S1 level, while T2WI showed increased T2 signal.	none	n/a	Discitis	Initially treated with prednisone, methotrexate, sulfasalazine, and hydroxychloroquine due to erroneous diagnosis of rheumatoid arthritis. When correct diagnosis was realized these were discontinued and was started on i.v. ceftriaxone. Was discharged with 3-month course of i.v. ceftriaxone.	Complete resolution of arthritis, marked improvement of back pain, normal inflammatory markers, and resolution of discitis on repeat MRI at 3 months follow-up.
##	Germany	Eisenberg et al.	59-year/male	Snake keeper who bred rats for snake food.	History: 15 days of fever and arthralgia without rash. Inability to stand and acute progressive onset of dyspnea. On exam: T 39 °C. Was initially sedated and placed on ventilator. With discontinuation of sedation, exam showed cervical pain, flaccid tetraplegia, sensitivity at the T4 level. Knees and left wrist swollen with joint effusions.	WBC 15 (predominantly neutrophils); C-reactive protein 125 mg/L.	C5–T1 vertebrae	16S rRNA gene sequencing from synovia	Blood cultures showed negative results. Culture-negative inflammatory liquid and uric acid crystals found in joint effusions.	Fat-saturated, contrast-enhanced T1-weighted MRI spine: Sagittal view of the cervical spine shows spondylodiscitis; epidural absess with C5–T1 compression.	none	n/a	Vertebral osteomyelitis and an epidural abscess with consecutive compression of the spinal cord (C5–T1)	Amoxicillin and cloxacillin	Not reported.
##	United Kingdom	Abusalameh et al	62-year/female	Denied history of rat bite; acknowledged exposure to live rats and rats droppings.	History: Four-days of diarrhea and vomiting followed by acute onset of diffuse hot, swollen joints with severe lower back pain. Hx of seropositive RA (positive anti-CCP), controlled with MTX and tocilizumab.	CRP 218 mg/l, creatinine 2.37	L5/S1 intervertebral disc	16S rRNA PCR	Knee, ankle, wrist, and L5/S1 disc needle aspirates grew a gram-negative organism.	MRI spine: edema of L5/S1 intervertebral disc.	none	n/a	Discitis	Initially on benzyl penicillin and clindamycin. Later changed to 12-week course of oral amoxicillin and clindamycin.	Disc edema improvement after weeks of antibiotic treatment.
##	Portugal	Pena et al.	75-year/female	Rat bite	History: Four-day history of fever, myalgias, headache. On exam: Subfebrile, hypotensive, incised wounds on two fingers of left hand. Neck stiffness. On day 3 of admission patient developed worsening neck pain and quadriparesis.	WBC 14,670/μL (86.3% neutrophils), CRP 334 mg/dL, normal LP	C5, C6, and C7 vertebrae, left SC joint	16S rRNA PCR and Sanger sequencing	Two blood cultures (BD BACTEC Plus Aerobic/F medium) grew gram-negative bacteria after 3 days incubation.	Normal CT brain. MRI T2WI: high signal intensity in C5, C6, and C7 vertebrae with meningeal enhancement and high signal intensity the left SC joint, consistent with diagnosis of vertebral osteomyelitis and septic arthritis	none	n/a	Vertebral osteomyelitis and septic arthritis	Empirically treated on day 1 of hospitalization with i.v. ceftriaxone (2 g/day); completed 26 days of i.v. ceftriaxone followed by 8 months of oral amoxicillin-clavulanate after discharge.	Complete resolution of neck pain and tetraparesis.

This case underscores the importance of obtaining a thorough zoonotic exposure history and maintaining a broad differential that includes RBF when considering potential causes of discitis and osteomyelitis.

## Data Availability

Not applicable.

## Abbreviations

RBF: Rat bite fever
MIC: Minimum inhibitory concentration
TEE: Transesophageal echocardiography
MALDI-TOF: Matrix-assisted laser desorption/ionization

## References

[1] Eisenberg T, Ewers C, Rau J, Akimkin V, Nicklas W (2016). Approved and novel strategies in diagnostics of rat bite fever and other *Streptobacillus* infections in humans and animals. Virulence.

[2] Eisenberg T, Nicklas W, Mauder N, Rau J, Contzen M, Semmler T, et al. Phenotypic and genotypic characteristics of members of the genus *Streptobacillus*. PLoS One. 2015;10(8) Cited 2021 Feb 28. 10.1371/journal.pone.0134312 PMC4529157 26252790

[3] Elliott SP (2007). Rat bite fever and *Streptobacillus moniliformis*. Clin Microbiol Rev.

[4] DuBray KA, Glaser CA, Cherry J, Demmler-Harrison GJ, Kaplan SL, Steinbach WJ, Hotez PJ (2019). *Streptobacillus moniliformis* (rat-bite fever). Feigin and Cherry’s textbook of pediatric infectious diseases.

[5] Royer N. The history of fancy rats: American Fancy Rat & Mouse Association; 2015. Available from: https://www.afrma.org/historyrat.htm. Cited 2020 May 30

[6] Abusalameh M, Mahankali-Rao P, Earl S (2018). Discitis caused by rat bite fever in a rheumatoid arthritis patient on tocilizumab – first ever case. Rheumatology.

[7] Pena MER, Jordão S, Simões MJ, Oleastro M, Neves I. A rare cause of vertebral osteomyelitis: the first case report of rat-bite fever in Portugal. Rev Soc Bras Med Trop. 2020;53. 10.1590/0037-8682-0328-2019. 10.1590/0037-8682-0328-2019 PMC7083378 31859955

[8] Nei T, Sato A, Sonobe K, Miura Y, Takahashi K, Saito R (2015). *Streptobacillus moniliformis* bacteremia in a rheumatoid arthritis patient without a rat bite: a case report. BMC Res Notes.

[9] Adizie T, Gayed M, Ravindran J (2014). Rat bite fever causing septic arthritis and osteomyelitis in a young man. Rheumatology.

[10] Dubois D, Robin F, Bouvier D, Delmas J, Bonnet R, Lesens O, Hennequin C (2008). *Streptobacillus moniliformis* as the causative agent in spondylodiscitis and psoas abscess after rooster scratches. J Clin Microbiol.

[11] Akter R, Boland P, Daley P, Rahman P, Al Ghanim N (2016). Rat bite fever resembling rheumatoid arthritis. Can J Infect Dis Med Microbiol.

[12] Sato R, Kuriyama A, Nasu M (2016). Rat-bite fever complicated by vertebral osteomyelitis: a case report. J Infect Chemother.

[13] Flannery DD, Akinboyo I, Ty JM, Averill LW, Freedman A (2013). Septic arthritis and concern for osteomyelitis in a child with rat bite fever. J Clin Microbiol.

[14] Eisenberg T, Poignant S, Jouan Y, Fawzy A, Nicklas W, Ewers C (2017). Acute tetraplegia caused by rat bite fever in snake keeper and transmission of *Streptobacillus moniliformis*. Emerg Infect Dis.

[15] Kache PA, Person MK, Seeman SM, McQuiston JR, McCollum J, Traxler RM (2020). Rat-bite fever in the United States: an analysis using multiple national data sources, 2001–2015. Open Forum Infect Dis.

